# Supplementary material for: Initial functional disability as a 1-year prognostic factor in geriatric patients hospitalized with SARS-CoV-2 infection
Source: PLoS One. 2023 Jul 27;18(7):e0289297. doi: 10.1371/journal.pone.0289297 (PMC10374042; doi:10.1371/journal.pone.0289297)
Supplement: Attachment — Submitted filename: Rev. Comments.pdf [file pone.0289297.s001.pdf]

Dear Editorial Office
PLOS One
Greeting, have a good day. Enclosed you will find kindly comments concerning **PONE-D-22-32169** manuscript.
December 8th 2022

Title:
Initial functional disability as a 1-year prognostic factor in geriatric patients hospitalized for SARS-CoV-2 infection

Corresponding Author: Olivier BRIERE

Dear Editor
Greeting,
Thanks for giving me opportunity to revise this piece of research work for your respective journal.

This manuscript attempted to verify Initial functional capacity which is considered an important prognostic marker in older patients hospitalized for COVID-19. The rationale and study design are suitable and good enough to support the author's hypothesis. However, some points need to be revised to be further considered for publishing.

Comments
Major Comments
First of all the authors depend on grouping of cases into two groups according to 1 year mortality (No and Yes) which cause misleading and the grouping is not clear
It was better to divide your groups according to GIR (GIR score ≤ 3 OR< 3)

Abstract
1- Line 51: "All-cause mortality was collected" did the authors collect only mortality? Or both morbidity & mortality?

Introduction
1- Line 86 what is AGGIR?

Methods
1- Please deliver an explanation about reasons of chosen old patients. Is that information makes any change or add significance idea from authors point of view to the manuscript? And if adult patient may show the same results or not?
2- Sample size is 97 patients which was small (All patients between March and June) why the authors didn`t extend the duration of the study? please provide an explanation for this
3- Authors should provide an explanation for why they didn`t tried to follow up their cases more than just the telephone follow up to serve their research results?
4- Authors should provide an explanation for why the duration of hospital stay not taken in consideration to serve their research results? and if all patient pass through the ICU at any time during their treatment curriculum?

Results

1- Please deliver an explanation about if there is a relation between COVID19 and malignancies as a comorbidity or malignancies are considered an individual cause of morbidity in those old aged, hospitalized patients?

Discussion

1- Please add a brief explanation about the meaning of qSOFA?
2- The authors define disability in their discussion but without providing the readers about most common disabilities the detected during their study, please explain
3- What are the authors recommendations to overcome multiple limitation mentioned in their manuscript?

Minor comments
Abstract

1- Sample size should be mentioned in the method section

Introduction

1- Line 81: reference missed

Methods

1- Please provide subtitles
2- Are there any excluded cases? Reasons and number of excluded cases should be mentioned in detail in the method

Results

Line 153: 88.0+5.4 years, please correct
Line 165: n=96 please correct

Discussion

1- Line 216: [19] italic, please correct
2- Line 242: reference missed

References

Reference 16: begin with "for the VIP2 study group "what did the authors mean?

Finally, the manuscript parts, Introduction, Methods, Results, Tables, and all illustration are done in very good way. I think once authors fix the above comments. The work will be ready to be published in your esteemed journal.

Best wish's
Sincerely
Dr. Eman Abdallah
Professor of forensic medicine and Toxicology
