# Supplementary material for: Initial functional disability as a 1-year prognostic factor in geriatric patients hospitalized with SARS-CoV-2 infection
Source: PLoS One. 2023 Jul 27;18(7):e0289297. doi: 10.1371/journal.pone.0289297 (PMC10374042; doi:10.1371/journal.pone.0289297)
Supplement: Attachment — Submitted filename: Rev. Comments R1 - Copy.docx [file pone.0289297.s003.docx]

**Dear Editorial Office**

PLOS One

Greeting, have a good day. Enclosed you will find kindly comments concerning **PONE-D-22-32169R1** manuscript.

July 14^th^ 20223

**Title:**

**Initial functional disability as a 1-year prognostic factor in geriatric patients hospitalized**

**with SARS-CoV-2 infection**

**Corresponding Author:** Olivier BRIERE

***Dear Editor***

***Greeting,***

***Thanks for giving me opportunity to revise this piece of research work for your respective journal.***

This manuscript attempted to verify Initial functional capacity which is considered an important prognostic marker in older patients hospitalized for COVID-19. The rationale and study design are suitable and good enough to support the author’s hypothesis.

**Comments**

The manuscript became suitable and fit well your esteemed journal

Decision **Accepted** with no further comments

***Best wish’s***

***Sincerely***

***Dr. Eman Abdallah***

***Professor of forensic medicine and Toxicology***
